# Supplementary material for: Identification of Language-Induced Mental Load from Eye Behaviors in Virtual Reality
Source: Sensors (Basel). 2023 Jul 25;23(15):6667. doi: 10.3390/s23156667 (PMC10422404; doi:10.3390/s23156667)
Supplement: Supplementary file 1 [file sensors-23-06667-s001.zip › sensors-2425717-supplementary.pdf]

Virtual Reality Language Booth

– Supplemental Information –

① Automatic VR introduction to the experiment

First, participants did not see the scene and just read some text in front of a gray back-ground. To avoid any hassle with controllers, participants were asked to wink to proceed. Texts were displayed in Japanese and English, both were checked by high-level speakers.

1. **Welcome**
   実験のご協力ありがとうございます。HMD を装着してください。（イヤーパッドも下ろしてください。）完了したら、ウインクをしてください。
   Welcome! Please adjust the HMD. (Don't forget to lower the earpods.) Once you are ready, just perform a wink!
2. **Adjusting initial standing position**
   ここからはウインクで次に進むことができます。ではマークの上に立ってください。【完了したら、ウインクをしてください。】
   You can always proceed by performing a wink. Confirm that you are standing on the marked spot. (Please wink to proceed.)
3. **Explaining (inbuilt) eye calibration**
   アイトラッキングのキャリブレーションを行います。青い点を目で追い続けてください。このキャリブレーションが不十分な場合、アプリケーションがうまく動作しないことがあります。【完了したら、ウインクをしてください。】
   Eye tracking will now be calibrated. Please follow the blue dot at all times! You will otherwise have problems interacting with the experiment. (Please wink to proceed.)
4. **Inbuilt eye calibration of HTC Vive Pro Eye is performed**
5. **Explaining the aim of the experiment**
   実験では、電話が４回かかってきます。電話から音声が流れるので、その音声をよく聞いてください。その時、その音声内容が流れた順番も覚えてください。アンケートでは「1 人目の通話相手」などと指示して質問します。【よろしければウインクをしてください。】
   In this experiment, you receive 4 phone calls. Just try to understand what is being talked about. However, please also remember the order. The questionnaire will refer to e.g. "the first speaker". (Please wink to proceed.)
6. **Explaining (custom) audio setting**
   音の大きさを設定します。マイナスマークに視線を向けると音は小さくなり、プラスマークに視線を向けると音が大きくなります。聞こえやすい大きさに調節してください。【完了したら、ウインクをしてください。】
   Audio volume will now be calibrated. Look at the minus or plus sign to raise or lower the volume. Please set it to a comfortable level. (Please wink to proceed.)
7. **Participants used gaze to adjust the audio volume (gazing at +/- signs)**
8. **Explaining (custom) eye tracking verification procedure**
   アイトラッキングの精度を確認します。必ずクロスヘアを目で追い続けてください。【完了したら、ウインクをしてください。】
   Eye tracking will now be verified.Please follow the crosshair at all times! (Please wink to proceed.)
9. **Participants followed a crosshair making some predefined moves**
10. **Reminder to not close the eyes intentionally**
    実験の準備ができました！実験中はできるだけ目は閉じないようにしてください。ではバーチャルな世界で臨場感を体験してください！【完了したら、ウインクをしてください。】
    Congratulations, you are ready now! Try to experience presence in the virtual world. Please don't close your eyes except for regular blinking. (Please wink to proceed.)
11. **Scene is displayed, and the phone starts ringing**

② Transcript of speech samples used

The following samples were taken from natural, native language and selected using an intuitive sense of difficulty. Formally categorized training material is often unnatural and was not considered for this study. The translations were checked by high-level speakers of Japanese.

1. **JP-Familiar**
   (Not publicly available, a very similar example can be found here.)
   同窓会の役割なんですけれども、ここにありますように、大学っていうのは、その後、生涯にわたる人と人のネットワークを作る場所でもあります。で、そのあとですね、皆さんは社会に出ていくことになるんですけれども、ここの大学院で培ったヒューマンネットワークっていうのは、そのあと、年を重ねるごとにすごくありがたく思うものです。なので、社会で活躍する皆さんの先輩たちとたくさん知り合ったり、もちろん、同じ学年の人たちと友達になったり、たくさんいい出会いをしてくれたらいいかなと思っています。同窓会は、ＯＢ・ＯＧ・同じ学年の皆さんの人と人のつながりを強める、そういう連携を強化するところをサポートする役割がございます。
   This is about the role of the alumni association. As you can see here, university is a place to make lifelong connections. And after students leave university, participate in society and get older, these connections are then often regarded as something extremely valuable. I hope you will be able to meet and make friends with many previous students and also students from the same year through this. The alumni association has the role to strengthen these connections and help with keeping up an active exchange between former and current students.
2. **JP-Unfamiliar**
   (From Psycho-Pass: The Movie, at around 0:27:30, a very similar example can be found here.)
   あらゆる秩序、あらゆる価値観が混沌の中に消え去った。今となっては、大多数の人類がただ欲望に駆られるがままの動物じみた生活を余儀なくされている。いずれにせよ、人が人らしく思想と尊厳を保てる環境など、どこにもない。そんな中で、自らの価値基準だけを道しるべに運命を切り開いていける意志と行動の力、ことさらに輝いて見えるものだろう。とりわけ、犯罪すら辞さないほど苦境に追い詰められた人間にとっては、な。
   Any kind of order, any sense of values dissolved into chaos. Right now, most of mankind live like the animals, simply chased by their desires. Anyway, an environment where ideals and dignity can be preserved does not longer exist on this earth. Within all this, it will be even more remarkable to only act upon one’s own values and have the will to follow destiny in one’s own right. Especially to people who have been put under circumstances where crimes are daily fare.
3. **EN-Familiar**
   (From here, until around 00:58.)
   Oh, hi everyone! Here, in Washington DC, the weather changes often. On one day, it is cold and windy, but the next day, it is warm and sunny! So everyday, I check the forecast. Hello, phone? What is today’s temperature? Today, it is 18 degrees. 18 degrees! That is cold! 18 degrees Celsius. Oh, Celsius. That is 65 degrees Fahrenheit. That’s warm! Yes Anna, it is warm.
4. **EN-Unfamiliar**
   (From here, until around 34:00.)
   A game for a little kid has to be immediately rewarding. And then, the game shades into real life. But as the game shades into real life, what happens is that the rewards are deferred. And you get more and more disciplined at not being immediately rewarded, like when you are learning to read or play the piano, for the long-term goal. The thing about video games is that the do require the development of skill, but the immediate reward is built in along with the delayed reward, because otherwise, the game wouldn't be fun for someone who's learning. So, the problem is that a lot of real-life games aren't necessarily fun while you are learning them. Cause you have to attain a certain level of mastery, and that requires discipline.

③ Full contents of the post-questionnaire

The following questions were answered immediately after the VR experience, seated at a PC.

1. ユーザ ID (実験終了時に表示されていた値)
   User ID (As displayed at the end of the experiment)
2. 性別
   Gender
3. 年齢
   Age
4. 一人目の通話相手が話した具体的な内容もしくはその趣旨
   Contents and suspected context of what speaker number 1 talked about
5. 一人目の通話相手が話した言語 (どの程度の確信があるかを明記してください)
   Language that speaker number 1 spoke in (Guess? How certain?)
6. 一人目の通話相手が話した内容をどの程度理解できましたか.
   How well do you feel that you understood speaker number 1?
7. 二人目の通話相手が話した具体的な内容もしくはその趣旨
   Contents and suspected context of what speaker number 2 talked about
8. 二人目の通話相手が話した言語 (どの程度の確信があるかを明記してください)
   Language that speaker number 2 spoke in (Guess? How certain?)
9. 二人目の通話相手が話した内容をどの程度理解できましたか.
   How well do you feel that you understood speaker number 2?
10. 三人目の通話相手が話した具体的な内容もしくはその趣旨
    Contents and suspected context of what speaker number 3 talked about
11. 三人目の通話相手が話した言語 (どの程度の確信があるかを明記してください)
    Language that speaker number 3 spoke in (Guess? How certain?)
12. 三人目の通話相手が話した内容をどの程度理解できましたか.
    How well do you feel that you understood speaker number 3?
13. 四人目の通話相手が話した具体的な内容もしくはその趣旨
    Contents and suspected context of what speaker number 4 talked about
14. 四人目の通話相手が話した言語 (どの程度の確信があるかを明記してください)
    Language that speaker number 4 spoke in (Guess? How certain?)
15. 四人目の通話相手が話した内容をどの程度理解できましたか.
    How well do you feel that you understood speaker number 4?
16. 母国語
    Your native language(s)
17. 第二言語 (最も親しみのある外国語)
    Your other languages (best first)
18. 全体として、あなたは「外国語能力を向上させたい」というモチベーションはどの程度感じていますか？
    How motivated do you generally feel to improve your foreign language skills?
19. 実験中に眼鏡をかけていましたか.
    Did you wear glasses beneath the VR headset?
20. 実験について何かコメントがありますか. 良かった点悪かった点何でも結構です.
    What do you think about the experiment? How fun was it? Did you encounter problems?
